# Shear Wave Elastography Adds to the Sensitivity of Cancer Diagnosis in AUS-Nuclear and AUS-Other Nodules > 1 cm, but It Cannot Replace TIRADS Assessment and Repeat FNA

**DOI:** 10.3390/cancers18172737

**Published:** 2026-08-23

**Authors:** Dorota Słowińska-Klencka, Bożena Popowicz, Joanna Duda-Szymańska, Mariusz Klencki

**Affiliations:** 1 Department of Morphometry of Endocrine Glands, Medical University of Lodz, 251 Pomorska Str, 92-213 Lodz, Poland; dorota.slowinska-klencka@umed.lodz.pl (D.S.-K.); bozena.popowicz@umed.lodz.pl (B.P.); 2 Department of Pathomorphology, Medical University of Lodz, 251 Pomorska Str, 92-213 Lodz, Poland; joanna.duda-szymanska@umed.lodz.pl

**Keywords:** thyroid cancer, elastography, SWE, FNA, AUS-nuclear, AUS-other

## Abstract

**Background/Objectives:** The aim of the study was to compare the effectiveness of SWE in AUS-nuclear and AUS-other nodules, including nodules with an unequivocal cytology (UC), taking into account the size of the nodules. **Methods:** We evaluated 100 AUS-nuclear and 131 AUS-other nodules with known results of EU-TIRADS, repeat FNA (rFNA), and histopathological examination, and 92 nodules with UC: 47 malignant and 45 benign. Two SWE measures were used: Ewhole—capturing the largest possible area of the nodule; and Estiffest—limited to its hardest part. **Results:** In the UC group, values of both SWE measures above the thresholds were a significant malignancy risk feature regardless of the nodule size, but only Estiffest in nodules > 1 cm was an independent malignancy risk feature. In both AUS subcategories, similar results were obtained only for nodules > 1 cm; but, even in this group, rFNA and EU-TIRADS had a higher accuracy than SWE in the differentiation of benign and malignant nodules, and no SWE measures were an independent malignancy risk feature. However, in the AUS-nuclear subcategory, where PTC predominates among cancers, adding Estiffest to the EU-TIRADS and rFNA assessment led to some increase in sensitivity with an acceptable decrease in specificity. In the AUS-other subcategory, where the profile of cancers is more diverse, a similar effect was obtained by the addition of Ewhole. **Conclusions:** SWE may complement rFNA and TIRADS in category III nodules > 1 cm, but the decision whether to apply SWE and which measures should be used depends on the pre-test probability of cancer and the expected frequency of PTC among cancers.

## Simple Summary

The reports on the usefulness of shear wave elastography (SWE) in the diagnostics of thyroid nodules are ambiguous. The aim of the study was to analyze two important issues that could affect the results obtained so far: (1) the selection of the nodules—with or without nodules with indeterminate cytology; and (2) the size of the nodules. Our results indicate that it is not reasonable to translate the conclusions about the effectiveness of SWE from the group of nodules with an unequivocal cytology to the group of nodules with an indeterminate cytology, particularly Bethesda category III. In this category, the effectiveness of SWE is insufficient and has no advantages over the repeat FNA and TIRADS class assessment. However, the addition of SWE to these methods increases the sensitivity for detecting cancers in nodules > 1 cm at the expense of a decreased specificity.

## 1. Introduction

The diagnosis of thyroid nodules is based on an ultrasound examination (US) and fine-needle aspiration biopsy (FNA). The US is used to select nodules for FNA, based on the assessment of ultrasound malignancy risk features [1,2,3]. These characteristics determine the assignment of the nodule to the appropriate class of the selected ultrasound risk stratification system, usually referred to by the acronym TIRADS. The combined assessment of the nodule’s TIRADS class and FNA category, formulated in accordance with the Bethesda System for Reporting Thyroid Cytopathology (BSRTC), is the basis for making decisions about surgical or conservative treatment [1,2,3]. Unfortunately, in a significant number of cases, the diagnostic conclusion formulated on the basis of these methods is ambiguous (BSRTC categories III, IV, and V). This leads to potentially unnecessary thyroid surgeries, which are performed not as an optimal mode of treatment, but to resolve diagnostic doubts. The greatest controversy concerns category III BSRTC—atypia of undetermined significance (AUS)—because this category is characterized by the highest variation in the related risk of malignancy between centers, ranging from 5 to 70% [4,5]. There are two subcategories distinguished within category III: AUS-nuclear—describing atypia of undetermined significance concerning cell nuclei, and AUS-other—referring to atypia of undetermined significance of another type [1]. According to many studies, including ours, the former of these subcategories presents a two-fold higher risk of malignancy than the latter, and it is also more often associated with papillary thyroid cancer (PTC) [4,5,6,7]. In the case of category III, repeat FNA (rFNA) is recommended, with the addition of expensive molecular tests when available. However, the results of rFNA often remain ambiguous [1,2,3]. Therefore, other methods to support the diagnosis of thyroid nodules are being sought. One of the most accessible among them is elastography (ES), a method based on ultrasonography.

Elastography is used to assess the stiffness of the nodule and, on this basis, to presumably predict the presence of cancer. There are two main approaches to performing the ES measurement: the quasi-static method, termed strain elastography (SE), and dynamic methods, including 2D shear wave elastography (SWE), point SWE (pSWE), and acoustic radiation force impulse (ARFI) imaging [8,9,10]. Dynamic methods use standardized acoustic impulses from the US transducer to induce minute tissue movements, resulting in transverse shear waves. The shear wave speed is then registered and translated into a quantitative measure of elasticity. The result is either specified in m/s units or transformed to kilopascals (kPa)—expressing tissue stiffness—using a formula comprising tissue density and propagation velocity [10,11,12,13]. The higher the measured velocity or propagation speed, the stiffer the tissue and the greater the risk of a pathological lesion in the examined tissue. The dynamic methods are recognized as more objective and less user-dependent than SE. However, data on their usefulness in diagnostics are ambiguous. The initial enthusiasm and expectations that these methods are superior to any conventional US markers [14] and may even replace conventional US or even FNA have diminished in recent years [12,15,16,17]. It has been found that the establishment of universally applicable diagnostic criteria for SWE, the most popular of the dynamic methods, remains an insurmountable challenge [18]. This is caused by two main reasons: firstly, the technical and methodological aspects (differences in the apparatus used and the way the measurements are made), as many authors point out [11,12,13]; and, secondly, the differences between the studied populations in terms of the frequency and type of pathological lesions of the thyroid gland [4]. These differences result both from epidemiological reasons beyond the control of the investigators (mainly the iodine supply) and from the way patients are qualified for the study. In our opinion, the latter factor has not been taken into account when comparing SWE analyses, at least not to an adequate degree. Thus, we decided to examine its significance by comparing the effectiveness of SWE in the group of nodules with an unequivocal cytological result and in the group of nodules from category III BSRTC, with a separate consideration for its subcategories. To our knowledge, the SWE measurements in AUS-nuclear and AUS-other nodules have not been compared so far.

Therefore, the aim of our study was to analyze the effectiveness of the SWE assessment separately for the AUS-nuclear and AUS-other nodules and compare it with the effectiveness in a group of nodules with an unequivocal diagnostic conclusion (benign lesion and malignant tumor). The secondary aim was to determine the effectiveness of the SWE in combination with the TIRADS and rFNA assessment in both types of AUS nodules, taking into account the size of the nodules.

## 2. Materials and Methods

### 2.1. Patients

The study was performed in 2023–2025 at an endocrine center that specialized in the ultrasound and biopsy diagnostics of the thyroid gland. Patients with a BSRTC category III nodule who were referred to the follow-up FNA were enrolled in the study if they did not meet any exclusion criterion. The exclusion criteria were as follows: age under 18 years, FNA of the nodule in the last 3 months, a history of thyroidectomy, lobectomy, ^131^I treatment, or radiation therapy of the neck area. Nodules with suboptimal quality of SWE images were also excluded—in particular, nodules located in the isthmus, nodules located very deep, nodules with eggshell calcification, nodules with macrocalcification that occupied more than 25% of the nodule, or nodules with a cystic component larger than 50% of the nodule’s volume. At this stage, 406 patients were enrolled in the study, 155 with AUS-nuclear nodules and 251 with AUS-other nodules (Figure S1). From this group, 231 patients were qualified for the analysis, all of them with the definite diagnosis established on the basis of postoperative histopathological examination: 100 patients with an AUS-nuclear nodule and 131 with an AUS-other nodule (Table 1). The decision about the surgical treatment was made by doctors from the endocrine outpatient clinic based on the results of routine examinations, including the results of rFNA (without molecular tests) and patient preferences.

Additionally, 150 patients with unequivocal cytology (UC), 50 with a diagnosis of thyroid cancer (category VI BSRTC) and 100 with a diagnosis of a benign lesion (category II BSRTC), were enrolled in the study as they did not meet any exclusion criterion. From this group, 92 patients were qualified for the analysis: 47 patients with thyroid cancer confirmed in postoperative histopathological examination and 45 patients with a benign nodule—in 15 cases confirmed histopathologically and in 30 cases with at least one more FNA re-diagnosis of a benign lesion.

### 2.2. Routine US, FNA, and Histopathological Examinations

All ultrasound examinations were performed using the Aplio α ultrasound system (Canon Medical Systems Europe B.V., Zoetermeer, The Netherlands) equipped with an 18LX7 matrix linear probe. To ensure uniformity, machine parameters were standardized by applying the same thyroid ultrasound preset for all scans. Classical ultrasound malignancy-risk features were assessed prospectively, immediately before each FNA procedure. A custom computer program was used to record detailed information about each examined nodule—its location, dimensions, and sonographic characteristics—into a dedicated database. In particular, features relevant to the European Thyroid Imaging and Reporting Data System (EU-TIRADS) were documented [3]. This system is routinely applied in our center to guide the selection of nodules for FNA.

EU-TIRADS distinguishes five categories: EU-TIRADS 1 corresponds to an ultrasound examination in which no thyroid nodule is detected. EU-TIRADS 2 (benign category) includes pure cysts and entirely spongiform nodules; FNA is not recommended. EU-TIRADS 3 (low-risk category) includes isoechoic or hyperechoic nodules without any high-risk features; FNA is recommended for nodules larger than 20 mm. EU-TIRADS 4 (intermediate-risk category) includes hypoechoic nodules without high-risk features; FNA is recommended for nodules larger than 15 mm. EU-TIRADS 5 (high-risk category) includes nodules presenting at least one high-risk feature—non-oval shape, irregular margins, microcalcifications, or marked hypoechogenicity; FNA is recommended for nodules larger than 10 mm.

In selected cases, based on patient preference or the referring physician’s decision, FNA was performed in nodules smaller than the size thresholds recommended for their respective EU-TIRADS category. It was frequently the case for nodules of EU-TIRADS 5 category, because our national guidelines recommend FNA for nodules > 5 mm in this category [19].

FNA was performed under ultrasound guidance and consisted of two aspirations of the selected nodule. The smears were fixed in 95% ethanol and stained with hematoxylin and eosin. FNA results were classified according to the Bethesda System [1], following the most recent 2023 edition of the system, in which these categories are defined as follows: category I—(ND), category II—benign (BN), category III—atypia of undetermined significance (AUS), category IV—follicular neoplasm (FN), category V—suspicious for malignancy (SM), and category VI—malignant (MN). All nodules classified as category III in the BSRTC were further assigned to one of its two subcategories: AUS-nuclear or AUS-other. AUS-nuclear nodules were diagnosed when any abnormal nuclear features—such as nuclear enlargement, irregular nuclear contour or shape, prominent nucleoli, abnormal chromatin pattern, grooves, and/or intranuclear cytoplasmic inclusions—were present in an otherwise benign-appearing smear, or when nuclear atypia was observed in a specimen containing only a limited number of cells. AUS-other nodules were diagnosed when the specimen demonstrated architectural atypia (trabeculae, microfollicles, or cell crowding), a predominance of oncocytic cells, lymphocytic atypia, or atypia not otherwise specified, but without sufficient features to establish a diagnosis of neoplasia. All cytological evaluations were performed by a pathologist with more than 20 years of experience in thyroid cytology and histopathology.

Histopathological examination was conducted according to standard procedures, and diagnoses were formulated in accordance with the WHO classification of thyroid tumors [20]. Reclassification of histopathological specimens to identify cases of non-invasive follicular thyroid neoplasm with papillary-like nuclear features (NIFTP) was not performed. For the purposes of this study, follicular-cell-derived low-risk neoplasms—including NIFTP, thyroid tumors of uncertain malignant potential, and hyalinizing trabecular tumors—were grouped together with malignant nodules. According to WHO criteria, these neoplasms are considered borderline tumors, morphologically and clinically intermediate between benign and malignant lesions, yet possessing an extremely low but present potential for metastasis [20].

### 2.3. Elastography

**Figure 1:**
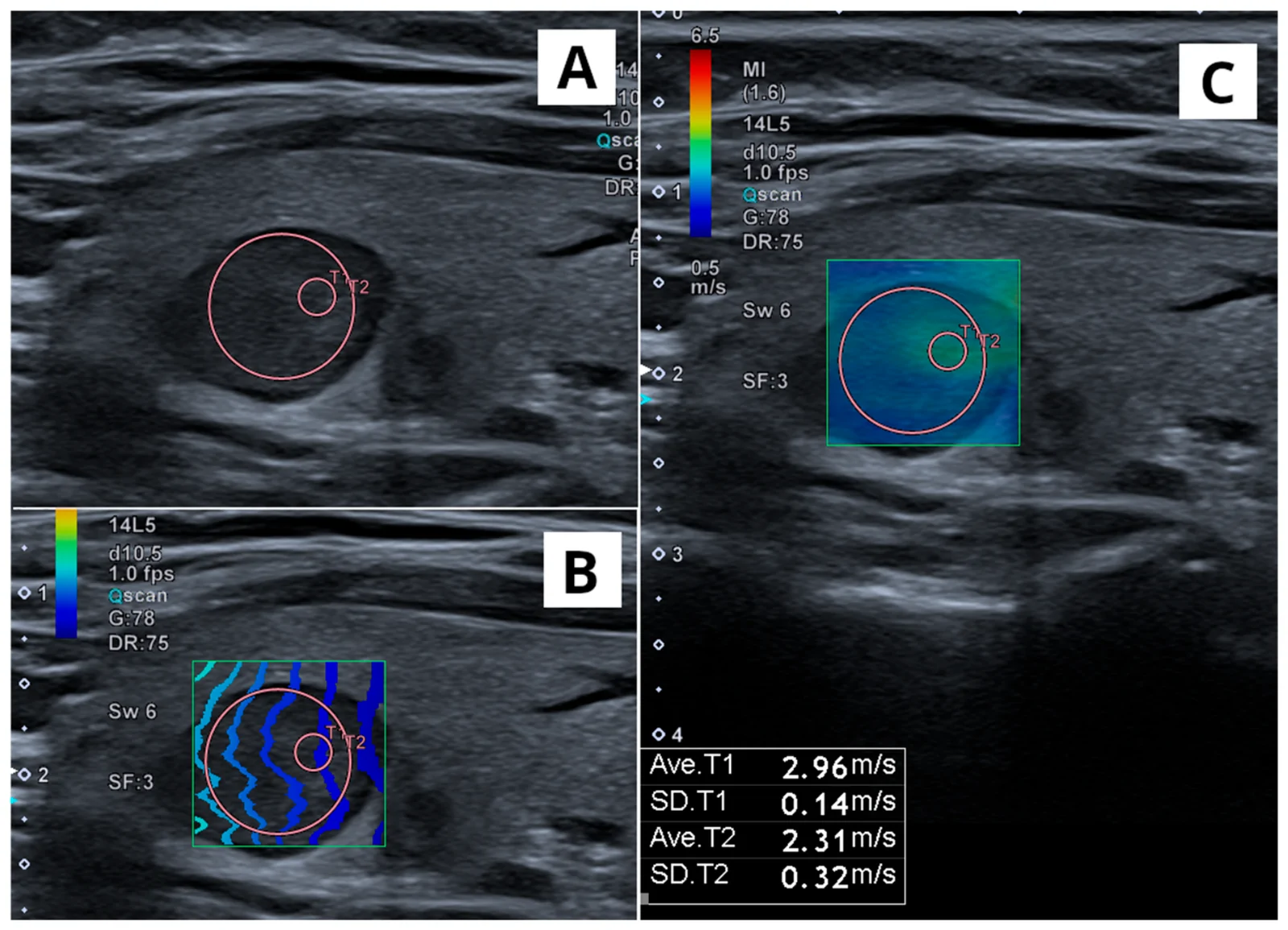
SWE examination of benign AUS-nuclear nodule, B-mode image (**A**); the map of shear wave propagation (**B**); and SWE measurement (**C**). T1 circle corresponds to Estiffest, and T2 to Ewhole.

SWE was performed in the longitudinal plane in order to minimize movement artifacts from the carotids. The probe was gently placed over the lesion to minimize compression artifacts [10,11,12,13]. Once a stable image had been acquired, the SWE sampling box—a square with sides measuring 1 cm—with the elasticity map (elastogram) was adjusted to encompass as much of the entire thyroid nodule as possible. In order to obtain a reliable image, the map of shear wave propagation provided by the ultrasound system was analyzed (Figure 1). The areas with non-parallel propagation of the waves were avoided.

Next, circular ROIs were placed within the elastogram, for which a summary of the stiffness data was automatically displayed. Two SWE measures were used as follows: (1) Ewhole, where a circular ROI was adjusted to match the lesion contours, capturing the largest possible area of the nodule; and (2) Estiffest, represented by a circular ROI with a diameter of 2 mm—it was placed at the hardest part of the solid nodules. The elastographic data were expressed as the mean value ± SD of shear wave speed (SWS) in m/s and as the mean value ± SD of elastic modulus (E) in kilopascals (kPa). All nodules were evaluated by a single operator with 10 years of experience in ultrasound elastography and over 20 years of experience in ultrasound diagnostics.

### 2.4. Statistical Analyses

The diagnostic performance of SWE was evaluated separately for AUS-nuclear nodules, AUS-other nodules, and UC nodules, based on their classification as benign or malignant. Cut-off values for both SWE parameters (Ewhole and Estiffest) were determined by analyzing the Youden index derived from receiver operating characteristic (ROC) curves. The effectiveness of these thresholds was assessed using sensitivity (SEN), specificity (SPC), accuracy (ACC), positive predictive value (PPV), negative predictive value (NPV), and the proportion of nodules exceeding the respective cut-off values. Odds ratios (ORs) with corresponding 95% confidence intervals (95% CI) were calculated using logistic regression.

Analogous to the SWE analysis, the diagnostic performance of EU-TIRADS and repeat FNA (rFNA) in distinguishing malignant from benign nodules was assessed using ROC curves, again separately for AUS-nuclear and AUS-other nodules. The overall effectiveness of all evaluated methods (SWE, EU-TIRADS, and rFNA) was compared using the area under the ROC curve (AUC). A similar comparison was made in the UC group for SWE and EU-TIRADS. On the basis of multivariate regression analysis, independent risk factors for malignancy in the studied groups were identified. Finally, we examined how the addition of SWE assessment to the EU-TIRADS class and rFNA assessment affected the diagnostic efficacy of differentiation between benign lesions and cancers. All analyses were performed separately for nodules ≤ 1 cm, which could be fully covered by the SWE sampling box, and nodules > 1 cm. The cut-off values were determined separately for SWS and E and expressed in m/s and kPa, respectively. The results of other statistical analyses were presented for SWS.

Statistical analyses were performed using Statistica (data analysis software system), version 13, TIBCO Software Inc. (2017), Santa Clara, CA, USA. Frequency distributions were compared using the chi-square test, with appropriate modifications depending on the number of analyzed cases. Continuous variables were compared between groups using either the Kruskal–Wallis test or the Mann–Whitney test. Differences between AUC values were assessed with the Z test. A significance level of 0.05 was adopted for all analyses. The study protocol was approved by the Local Bioethics Committee. All patients gave their informed consent for FNA.

## 3. Results

### 3.1. Characteristics of the Studied Groups

Table 1, Tables S1 and S2 in the Supplementary Materials present the relevant clinical data of the patients and characteristics of the thyroid nodules studied. The volume of nodules classified into individual groups and the percentage of nodules ≤ 1 cm did not differ significantly. The incidence of malignancy in the AUS-nuclear group was higher than in the AUS-other group (37.0% vs. 18.3%, *p* = 0.0014). The most common cancer in all groups was PTC; its incidence among malignant nodules was higher in the UC group than in the AUS-nuclear group (95.7% vs. 73.0%, *p* = 0.0031) and AUS-other (62.5%, *p* = 0.0003) (Table S1). The differences in this respect were more pronounced for nodules > 1 cm (UC: 95.0%; AUS-nuclear: 64.3%; AUS-other: 46.2%) than for smaller nodules (UC: 96.3%, AUS-nuclear: 78.2%; AUS-other: 81.8%). In all groups, cancers corresponded more frequently to class 5 EU-TIRADS than benign lesions, and their volume was significantly lower than the volume of benign lesions. In the case of cancers from both AUS groups, the most frequent outcome of rFNA was BSRTC category V, while, on the rFNA of benign nodules, category III or II were usually reported (Table S2). There were no significant differences in the mean age of patients among the groups, but, in the UC and AUS-nuclear groups, the mean age of patients with cancer was lower than the age of those with benign nodules. There were no significant differences between groups in the incidence of Hashimoto thyroiditis (HT), nor in the incidence of HT between patients with cancers and benign lesions in either group.

### 3.2. SWE Efficacy in UC, AUS-Nuclear, and AUS-Other Groups

In the AUS-nuclear and AUS-other groups, cancers had significantly higher values of SWE measures than benign nodules only in the case of nodules > 1 cm (Table 2).

For such nodules in the AUS-nuclear group, the cut-off levels for SWE measures were Ewhole—3.43 m/s (36.2 kPa) and Estiffest—4.31 m/s (55.90 kPa); and, in the AUS-other group, they were lower and amounted to 3.22 m/s (31.3 kPa) and 4.09 m/s (52.0 kPa), respectively (Table 3). In the AUS-nuclear group, the AUC and ACC were slightly higher (NS) for Estiffest than Ewhole (0.696 vs. 0.634 and 81.7% vs. 75.0%, respectively), with the same SEN (50.0%), and, in the AUS-other group, the AUC and ACC were slightly higher (NS) for Ewhole than Estiffest (0.752 vs. 0.697 and 83.8% vs. 81.3%), with a marked difference in SEN (respectively: 61.5% vs. 46.2%) (Table 3). The values of both SWE measures above the designated cut-off levels significantly suggested the malignancy of AUS-nuclear and AUS-other nodules > 1 cm in size (Table 4).

In the UC group, both SWE measures had significantly higher values in cancers than in benign nodules in both > 1 cm and smaller lesions (Table 2). The calculated cut-off levels for nodules > 1 cm were lower than for nodules ≤ 1 cm, with Ewhole: 3.40 m/s (36.3 kPa) vs. 4.28 m/s (56.0 kPa); and Estiffest: 4.19 m/s (53.3 kPa) vs. 4.36 m/s (57.6 kPa) (Table 3). The effectiveness of SWE measures measured by the AUC and ACC was slightly higher (NS) for Estiffest than for Ewhole irrespective of nodule size (nodules > 1 cm: 0.748 vs. 0.744 and 82.1% vs. 80.4%; nodules ≤ 1 cm: 0.805 vs. 0.753 and 77.8% vs. 72.2%, respectively) (Table 3). The increase in malignancy risk at those thresholds was significant for both SWE measures in both nodule size categories (Table 4).

### 3.3. Values of SWE Measures in Relations to Nodule Size

Benign nodules ≤ 1 cm had significantly higher Ewhole values in all groups than benign nodules > 1 cm (*p* < 0.05 in all cases) (Table 2). In the case of cancers, similar differences were observed only in the UC group (*p* < 0.05). In both AUS subgroups, the opposite trend was observed; the values of both SWE measures were slightly lower (NS) in cancers ≤ 1 cm than in larger cancers and significantly lower than in cancers ≤ 1 cm of the UC group (*p* < 0.0005 in all cases). In the case of benign AUS nodules, the values of both SWE measures were significantly lower than those observed in their UC counterparts (nodules ≤ 1 cm: *p* < 0.05; nodules > 1 cm: *p* < 0.005 in all cases).

### 3.4. Effectiveness of SWE, EU-TIRADS and rFNA

**Figure 2:**
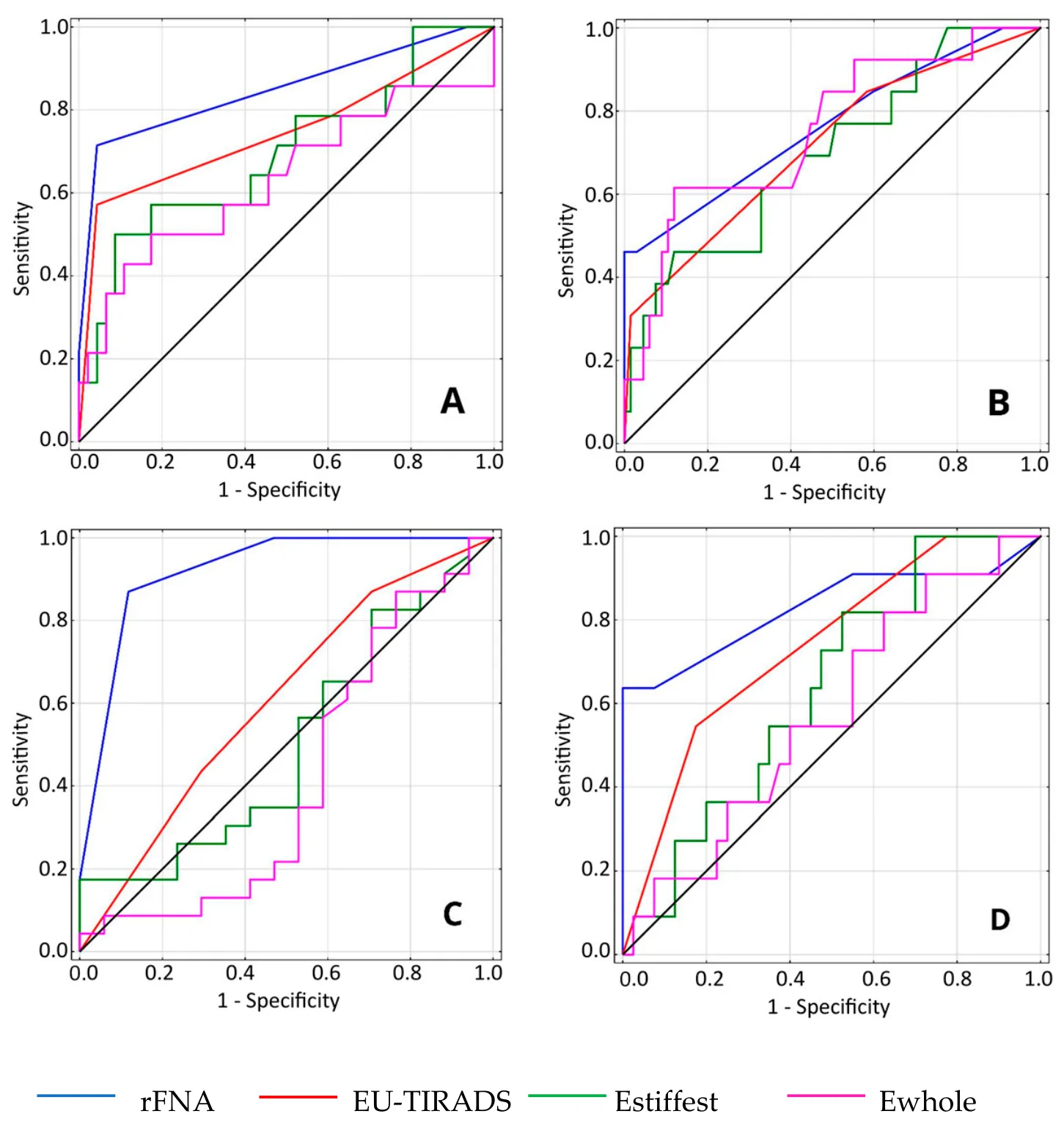
ROC curve analysis of repeat FNA, EU-TIRADS, Estiffest, and Ewhole evaluation in AUS-nuclear nodules > 1 cm (**A**); AUS-other nodules > 1 cm (**B**); AUS-nuclear nodules ≤ 1 cm (**C**); and AUS-other nodules ≤ 1 cm (**D**).

In both AUS groups, for both nodule size categories, rFNA had the highest ACC in the differentiation of benign and malignant nodules, followed by EU-TIRADS and the lowest ACC of SWE (Table 3). Similar relationships were observed for the AUC, with an exception for AUS-other nodules > 1 cm, where the AUC declined gradually from rFNA through Ewhole, EU-TIRADS, and down to Estiffest (Figure 2). Statistically significant differences in AUC were noted in the group of AUS-nuclear nodules > 1 cm: rFNA (0.849) vs. Estiffest (0.696, *p* = 0.0492) and vs. Ewhole (0.634, *p* = 0.0327); in the group of AUS-nuclear ≤ 1 cm: rFNA (0.921) vs. EU-TIRADS (0.710, *p* = 0.0112), vs. Estiffest (0.503, *p* = 0.0002), and vs. Ewhole (0.416, *p* < 0.0001); and, in the group of AUS-other nodules ≤ 1 cm: rFNA (0.830) vs. Ewhole (0.574, *p* = 0.0379). Multivariate regression analysis including SWE, EU-TIRADS, and rFNA showed that values of SWE measures exceeding the thresholds were not an independent cancer risk factor in any of the AUS subcategories. In both groups, for both nodule sizes, such an independent factor was at least category 5 of BSRTC in rFNA; and, in the AUS-other group for nodules > 1 cm, category 5 EU-TIRADS was also such a factor (Table 4).

When the regression analysis was limited to the ultrasound methods only, without rFNA, it showed that, in the AUS-other nodules > 1 cm, Ewhole values above the threshold were a risk factor for malignancy independently of the EU-TIRADS class (OR [95%CI]: 3.0 [1.3–6.9], *p* = 0.012).

In the UC group for nodules > 1 cm, an independent malignancy risk factor was, apart from EU-TIRADS class 5, with the Estiffest value above the threshold (Table 4). For UC nodules > 1 cm, the ACC and AUC were slightly higher (NS) for Ewhole and Estiffest than for EU-TIRADS (ACC: 80.4% and 82.1% vs. 76.8%; AUC: 0.744 and 0.748 vs. 0.725, respectively), while, for nodules ≤ 1 cm from the UC group, this relationship was inverse, and higher ACC and AUC values were achieved for EU-TIRADS than for Ewhole and Estiffest (ACC: 72.2% and 77.8% vs. 80.6%; AUC: 0.753 and 0.805 vs. 0.809) (Table 3).

### 3.5. Joint Assessment of SWE, EU-TIRADS, and rFNA in AUS-Nuclear and AUS-Other Nodules

For nodules > 1 cm in both the AUS-nuclear and AUS-other groups, the combined rFNA and EU-TIRADS evaluation had the highest ACC and the highest AUC (Table S3). Adding SWE to this set led to an increase in SEN by 7–8 percentage points in both AUS groups at the expense of a decrease in SPC by 8–10 percentage points. In the AUS-nuclear group, SEN maximization was achieved by the addition of Estiffest to EU-TIRADS and rFNA. Such a set yielded 85.7% SEN and 82.6% SPC, with an increase in the percentage of positive nodules from 25.0% to 33.3% and NPV maximization (95.0%) (Table 5).

In the AUS-other group, SEN maximization was achieved by taking into account Ewhole. Very similar results were obtained for the model based on the addition of Ewhole to the classical EU-TIRADS and rFNA assessment, and for the model including only Ewhole and rFNA or only Ewhole and EU-TIRADS. Those sets allowed achieving 61.5% SEN and 86.6–88.1% SPC with an increase in the percentage of positive nodules from 10.0% to 20.0–21.2% and the highest NPV (92.2%) (Table 5). No significant differences in AUC were found between those models.

In the case of nodules ≤ 1 cm (Table S4) in the AUS-other group, the inclusion of SWE was not justified due to a very large reduction in SPC and ACC along with a doubling of the percentage of positive nodules. On the other hand, in the AUS-nuclear group, the Estiffest was characterized by a low SEN but high SPC, and the addition of Estiffest to rFNA instead of EU-TIRADS increased the ACC by several percentage points, although the differences in AUC for the rFNA/Estiffest and rFNA/EU-TIRADS models were not significant.

## 4. Discussion

The lack of consistent conclusions is quite common among publications devoted to the usefulness of SWE in the diagnosis of thyroid nodules. In some meta-analyses, especially older ones, the authors have claimed that SWE is very accurate in distinguishing malignant and benign nodules and that it has an advantage over conventional US and/or TIRADS [21]. In other studies, researchers have drawn the somewhat softened conclusion that SWE is an essential tool that can complement ultrasound in clinical practice [18]. However, there are also studies in which the authors emphasize the suboptimal diagnostic accuracy of thyroid SWE despite the finding that malignant nodules have significantly higher quantitative SWE parameters than benign nodules [15,16,17].

**Figure 3:**
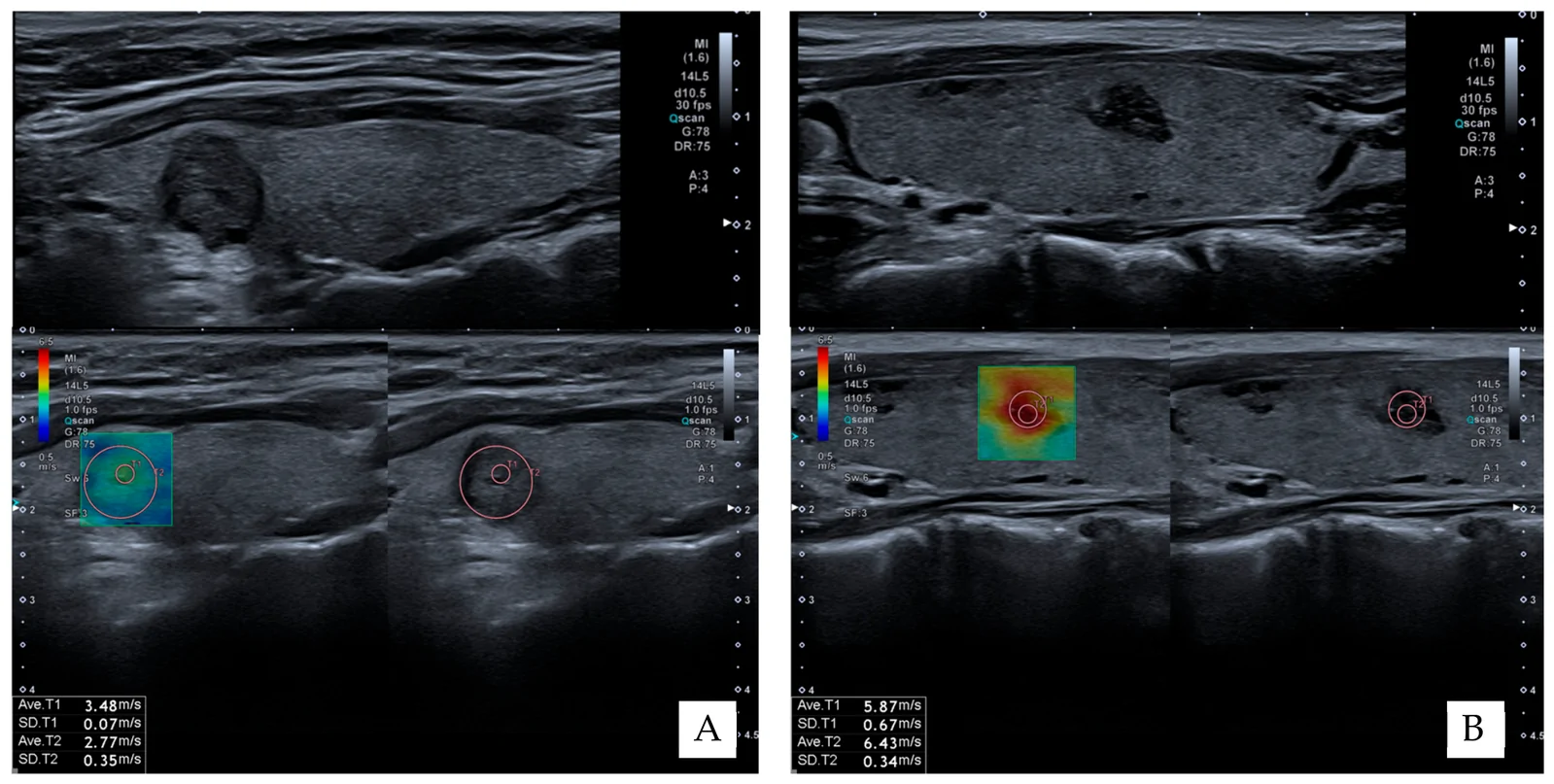
Classical US and SWE examination of two malignant nodules: (**A**) AUS-nuclear nodule B-mode image, EU-TIRADS 5 class—true positive, SWE measurements—false negative; and (**B**) category VI BSRTC nodule B-mode image, EU-TIRADS 5 class—true positive, SWE measurements—true positive.

In the context of these discrepancies, our study highlights two important issues that could affect the results obtained so far. One issue is related to the criteria for including nodules in the examination, and the other is associated with their size. Our results indicate that it is not reasonable to translate the conclusions on the effectiveness of SWE from the group of nodules with an unequivocal cytology to the group of nodules with an inconclusive cytology and, in particular, to nodules from category III BSRTC (see Figure 3 for an example of the different performance of SWE in these groups). In the former group, cancers are dominated by PTCs, which usually have not only a characteristic ultrasound and microscopic image, but also a higher stiffness in the elastographic examination than other cancers, in particular, follicular carcinomas [3,4,12,22]. Among AUS-nodules, there is not only a larger diversity of cancers, especially in the AUS-other subcategory, but also a higher incidence of non-classic subtypes of PTC, which show a lower stiffness than classic PTC [22,23]. As a result, in the case of nodules from category III, not only is the usefulness of assessing the classical ultrasound features of the malignancy risk lower, but the effectiveness of SWE is also impaired. The differences in comparison to the UC group also concern the cut-off values for SWE measures and the usefulness of individual SWE measures. In the case of the predominance of PTC among cancers (categories VI and AUS-nuclear nodules), Estiffest is more useful, while, in the case of nodules with a more diverse cancer profile (AUS-other nodules), Ewhole is more reliable. In addition, this issue is deepened by differences in the effectiveness of SWE resulting from the size of the nodules. They concern both AUS nodules and nodules with an unequivocal cytology, but, in the case of AUS nodules, the nodule size issue intensifies the problems resulting from the lower percentage of PTC among cancers. As a result, in both AUS groups, SWE showed any diagnostic value only in the case of nodules > 1 cm in size, and, even for these nodules, its ACC was lower than that of EU-TIRADS. None of the SWE measures was an independent malignancy risk factor in the AUS groups, although their values above the threshold significantly suggested the malignancy of nodules > 1 cm. In the case of nodules with an unequivocal cytology, values above the thresholds for both SWE measures were a malignancy risk factor for both small and larger nodules. However, only Estiffest was a risk feature independent of the EU-TIRADS class and only for nodules > 1 cm.

Our research also clearly indicates the advantage of rFNA over both ultrasound methods (EU-TIRADS and SWE) in both AUS groups. A positive rFNA result proved to be an independent risk factor for malignancy for both nodule sizes studied. The combined EU-TIRADS and rFNA assessments showed an over 80% ACC regardless of the AUS subcategory and nodule size (nodules > 1 cm, AUS-nuclear: 88.3%, AUS-other: 91.5%; nodules ≤ 1 cm, AUS-nuclear: 85.0%, and AUS-other: 80.4%). All other rFNA, EU-TIRADS, and SWE measure associations had a lower ACC. Only for the AUS-nuclear ≤ 1 cm nodules did the rFNA and Estiffest association (without EU-TIRADS) have a few-percentage-points-higher ACC. However, the isolated assessment of Estiffest, without classical ultrasound, should not be used in this group due to the very low SEN (<20.0%). Thus, SWE does not have the potential to replace the rFNA or TIRADS class rating of BSRTC category III nodules, but it may complement these methods to increase the SEN. For AUS-nuclear nodules > 1 cm, the inclusion of the Estiffest rating increases SEN by several percentage points with an acceptable reduction in SPC. On the other hand, in the case of AUS-other nodules > 1 cm, a better effect is obtained by attaching the Ewhole rating. For nodules ≤ 1 cm, the association of the SWE assessment with EU-TIRADS and rFNA led to a very large increase in false positive diagnoses and was not effective.

It is not easy to compare our results with other publications on the usefulness of SWE in the diagnosis of BSRTC category III nodules. To our knowledge, both subgroups of category III have not been studied separately so far; moreover, nodules from this category have been analyzed together with nodules from category IV [24,25,26,27,28,29,30] or with nodules from category IV and V [22,31,32]. These categories, like the two AUS subcategories, markedly vary in the pre-test probability of malignancy and the types of corresponding cancers [4,5]. Consequently, there is a lack of data to which we could directly relate our results. What is even more important, it is not surprising that the conclusions from publications on the usefulness of SWE in nodules with an ambiguous cytology, including BSRTC category III, are not consistent. In some studies in which nodules from category III and IV were analyzed together, the authors concluded that SWE had a higher value in predicting malignancy than classical ultrasound risk features and that SWE was able to select malignant nodules among those cytologically indeterminate [25,26,27]. In other publications, the authors, like us, pointed out that SWE could only complement the classical US by increasing the SEN of the diagnostics at the expense of SPC. Zhang et al. (2020) formulated such a conclusion by analyzing the combination of ACR TIRADS at category 5 with SWE in nodules of categories III–V [32]. Appanraj et al. (2024) evaluated ACR-TIRADS score 3 or 4 and Bethesda III–IV nodules and showed that SWE had a lower efficacy measured by the AUC than TIRADS, but it could be used as an adjunctive tool along with the routine ultrasound [29]. There are also studies in which the usefulness of SWE in the diagnosis of nodules with an ambiguous cytology has not been confirmed. Bardet et al. stated that SWE could not discriminate between benign and malignant tumors among thyroid nodules with a category III–V cytology [22]. Chambara et al. (2022) showed that, for cytologically equivocal nodules (category III–IV), the combination of EU-TIRADS with SWE resulted in a significant reduction in SEN with an increased SPC and it was not optimal for discriminating between benign and malignant nodules [28]. Celletti et al. (2021) concluded that the combination of SWE + K-TIRADS did not increase the diagnostic efficacy of K-TIRADS for category III–IV nodules [30]. Swan et al. (2019), by analyzing nodules from all categories of BSRTC, where PTCs accounted for only 65% of cancers, showed that SWE measures showed a huge overlap between malignant and benign nodules, and, consequently, a low diagnostic ACC in the prediction of thyroid malignancy [15]. They reached similar conclusions when they limited the analysis to nodules from category III–V of the BSRTC.

Opinions on the usefulness of particular SWE measures are also divergent and this applies not only to nodules with an ambiguous cytology. Any comparison is hampered not only by methodological differences, but even by the use of the same terms to describe different indicators measured. In some studies, the mean, maximum, and minimum stiffness of the nodule were determined in one ROI and different conclusions were obtained with such measurements: a higher usefulness of the Emax rating than Emean [33,34,35], the advantage of the Emean rating over Emax [36], the lack of differences between the above SWE measures [17], or the advantage of the standard deviation of elasticity indices [28]. In other analyses, as in our study, the average stiffness of the largest possible part of the nodule (usually denoted as Ewhole or Emean) and the average stiffness of a small nodule fragment with the highest stiffness (usually denoted as Estiffest or Emax, usually 1–2 mm in diameter) were measured. We did not find significant differences in the AUC between the SWE measures in any of the groups. However, we observed, as already mentioned, that, the higher the percentage of PTC among cancers, the higher the effectiveness of combining the Estiffest measure with EU-TIRADS, and, with the decrease in the PTC percentage, the importance of the combination of the Ewhole rating with EU-TIRADS increased. Perhaps for non-PTC cancers, it is less advantageous to search for the areas of the highest stiffness that may be related to the presence of microcalcifications, which are typical of PTC. Similarly, Wang et al. (2017), who analyzed a group of cancers composed almost exclusively of PTC, showed that Emax with SWS measurement is the strongest independent predictor for thyroid malignancy [37]. In turn, Qi et al. (2023), in a similar analysis, obtained better results for the assessment of Ewhole [38].

Our observations have also indicated that Ewhole is more susceptible to the influence of the nodule size. The Ewhole values for benign nodules ≤ 1 cm were higher than for larger nodules, and this was true for all studied groups, with no such differences for Estiffest. Perhaps what we treated as a potential advantage in the evaluation of small nodules—that is, the ability to assess the entire lesion with a bigger ROI—turned out to be a disadvantage. Covering an entire nodule with elasticity measurements reduces the chance of avoiding areas containing artifacts with a high, false-positive stiffness. This was probably one of the reasons for the lack of effectiveness of SWE in the group of small AUS nodules. The second reason was the tendency of small malignant AUS nodules to present a stiffness lower than the stiffness found in larger AUS nodules or in small malignant nodules unequivocally recognized in FNA. Other authors have also indicated that malignant nodules < 1 cm may present a low stiffness, thereby resulting in a poor discrimination from benign nodules using SWE [39].

Several previous analyses were devoted to assessing the effectiveness of SWE depending on the nodule sizes, but nodules with an inconclusive cytology were usually not examined separately in this context. In some of the studies, there was no relationship between the value of SWE measures and the size of the nodules [40]. There were no differences in the diagnostic efficacy measured by AUC between nodules < 1 cm and larger either [38]. In other studies, the authors—similarly to us—showed that the effectiveness of SWE was lower for nodules < 1 cm [37,41]. Xue et al. (2022) found that, in the case of nodules < 1 cm, SWE was predisposed to produce false negative results [42]. Wang et al. (2018) found that the differences in the mean elasticity in the size groups (nodules < 10; 10–20; >20 mm) were not statistically significant for malignant or benign nodules, but, when combined with SWE, US yielded a higher sensitivity for nodules > 1 cm and a higher specificity for nodules < 1 cm [43]. In turn, Gao et al. (2022) showed that adding SWE to the TIRADS assessment increased the diagnostic efficacy more noticeably for nodules < 1 cm than larger ones [34]. Duan et al. (2016) showed that conventional US combined with SWE had a high efficacy in the diagnosis of papillary microcarcinomas [44]. Bora Makal et al. (2021) compared nodules < 20 mm and larger ones and observed that the diagnostic performance of SWE decreased when the nodule diameter increased [45]. With regard to nodules with an ambiguous cytology, Chambara et al. (2022) analyzed the categories III–IV BSRTC together and showed that SWE in combination with EU-TIRADS was diagnostically efficient in discriminating nodules > 1 cm, but the SWE metric values did not differ significantly between malignant and benign nodules < 1 cm [28]. Therefore, they obtained results similar to ours. In turn, Celletti et al. (2021)—also in the group of nodules of category III–IV—found a higher SEN of SWE in nodules < 1 cm when compared to larger nodules [30].

This study has several limitations arising both from its design and from the characteristics of the examined patient population. Together, these factors make it difficult to directly generalize our findings to all category III nodules diagnosed in other centers.

Among the design-related limitations, several points should be highlighted: (a) The selection of category III nodules—only nodules treated surgically and therefore with available postoperative histopathological diagnoses were included. This approach ensures the certainty of the final diagnosis but increases the pre-test probability of malignancy, particularly in AUS-other nodules, and reduces the overall number of nodules available for analysis. (b) The exclusion criteria—nodules were excluded based on the location, presence of macrocalcifications, or a large cystic component, reflecting the fact that SWE cannot be reliably applied in certain lesions. (c) The subdivision of nodules by size and cytological subcategory—although this strategy enabled several important observations, it further reduced the size of the study groups. (d) The single-center design and use of specific equipment—all examinations were performed in one center using a particular ultrasound system, making the numerical SWE cut-off values specific to these technical conditions. (e) The classification of borderline tumors—we included borderline neoplasms in the malignant group, whereas other studies classify them as benign or exclude them entirely. As shown in our previous work, the impact of this classification is most pronounced in the AUS-nuclear subcategory [7]. (f) The lack of detailed histopathological data—information on PTC subtypes and on the detailed histopathological features of benign lesions (fibrosis, calcification, hyalinization, etc.) was unavailable. This limits the interpretation of some findings, including the unexpected observation that benign category III nodules exhibited a lower stiffness than benign nodules in the UC group.

Beyond factors related to study design, several characteristics of our patient population may have influenced the results: (a) A past iodine deficiency—a portion of our older patients were exposed to iodine deficiency in earlier decades, contributing to a relatively high prevalence of nodules with architectural atypia and frequent surgical treatment due to goiter size rather than alarming rFNA or ultrasound findings. The predominance of AUS-other over AUS-nuclear nodules observed in our cohort is typical for European populations, whereas, in countries with a long-standing iodine sufficiency, such as Korea and Japan, the proportions are usually reversed [4,5]. (b) The endocrine-center profile—as shown in our previous studies, endocrine centers tend to have a higher proportion of non-neoplastic lesions than oncological centers. In such patients, atypical features may more often be related to Hashimoto’s thyroiditis (HT), hyperthyroidism, antithyroid medications, or radioiodine therapy. Although the HT frequency did not differ significantly between groups, there was a tendency toward a higher HT prevalence in category III nodules, consistent with the literature indicating that HT increases the likelihood of ambiguous FNA results. HT typically increases thyroid stiffness due to parenchymal fibrosis [46], but, in early disease stages, the increased vascularity may reduce parenchymal stiffness. The influence of HT on SWE clearly warrants further investigation, ideally with a separate assessment of the background parenchymal stiffness and consideration of the disease stage. Despite these uncertainties, the lower stiffness observed in benign AUS nodules compared with benign nodules unequivocally diagnosed by FNA should not diminish the diagnostic utility of SWE; in fact, it might have facilitated distinguishing benign AUS nodules from malignant lesions.

## 5. Conclusions

In summary, within BSRTC category III nodules, the diagnostic performance of SWE in distinguishing benign from malignant lesions is limited and does not surpass that of rFNA or TIRADS classification. Importantly, SWE does not provide a stiffness-based metric that independently predicts malignancy in AUS nodules beyond what is already achieved by TIRADS and rFNA. Therefore, the TIRADS assessment—and, especially, rFNA—should remain the fundamental tools routinely used in the clinical management of AUS nodules.

SWE may, however, serve as a complementary method to the conventional ultrasound for nodules larger than 1 cm, provided it is applied with consideration of the pre-test probability of malignancy associated with each AUS subcategory in a given diagnostic setting. Selecting the appropriate diagnostic priorities is essential. For AUS-nuclear nodules, which carry a higher malignancy risk, maximizing sensitivity should be the primary goal. In contrast, for AUS-other nodules, which have a substantially lower malignancy risk, optimizing specificity is more important. Adding SWE to TIRADS increases sensitivity at the expense of specificity, making SWE most useful in AUS-nuclear nodules. In this subcategory, PTC predominates among cancers, which explains why Estiffest performs better than Ewhole. Conversely, in AUS-other nodules—characterized by a more heterogeneous cancer profile—SWE should be used more cautiously, and Ewhole appears to be the more effective metric.

It should also be emphasized that implementing SWE in any diagnostic center requires establishing center-specific cut-off thresholds. These thresholds depend on the unique characteristics of the local patient population, including the prevalence and types of thyroid pathology, as well as differences in the SWE acquisition algorithms and equipment-related technical factors. Further research on SWE is clearly warranted, particularly studies that separately evaluate AUS-nuclear and AUS-other nodules in larger cohorts. To date, our study is the first to address this issue in such detail.

## Figures and Tables

**Table 1:** Characteristics of the study groups in terms of age and gender of patients, coexistence of Hashimoto’s thyroiditis, volume of nodules, and their EU-TIRADS class.

	AUS-Nuclear				AUS-Other				Unequivocal Cytology			
	Benign 63	Malignant 37	*p*	All 100	Benign 107	Malignant 24	*p*	All 131	Benign 45	Malignant 47	*p*	All 92
Mean age of patients ±SD [year]	61.5 ± 14.1	51.9 ± 17.6	0.0035	57.9 ± 16.1	60.5 ± 13.5	59.0 ± 16.7	0.6555	60.2 ± 14.1	59.6 ± 15.5	51.3 ± 16.1	0.0053	55.3 ± 12.2
No. (%) of males	13 (20.6)	4 (10.8)	0.2067	17 (17.0)	15 (14.0)	4 (16.7)	0.7392	19 (14.5)	3 (6.7)	10 (21.3)	0.0870	13 (14.1)
No. (%) of patients with HT [No. (%)]	27 (42.9)	14 (37.8)	0.6222	41 (41.0)	46 (43.0)	10 (41.7)	<0.9057	56 (42.7)	12 (26.7)	17 (36.2)	0.3267	29 (31.5)
Mean volume of nodules ± SD [cm^3^]	3.6 ± 8.0	1.4 ± 4.5	0.0002	2.8 ± 6.9	2.9 ± 8.0	2.1 ± 7.1	0.0497	2.6 ± 7.8	3.2 ± 4.1	1.9 ± 6.4	0.0001	2.5 ± 5.4
No. (%) of nodules < 1 cm	17 (27.0)	23 (62.2)	0.0005	40 (40.0)	40 (37.4)	11 (45.8)	0.4429	51 (38.9)	9 (20.0)	27 (57.4)	0.0002	36 (39.1)
No. (%) of EU-TIRADS 3 nodules	23 (36.5)	6 (16.2)	0.0309	29 (29.0)	37 (34.6)	2 (8.35)	0.0218	39 (29.8)	15 (33.3)	2 (4.3)	0.0009	17 (18.5)
No. (%) of EU-TIRADS 4 nodules	33 (52.4)	13 (35.1)	0.0948	46 (46.0)	62 (57.9)	12 (45.8)	0.4781	74 (56.5)	24 (53.3)	12 (25.5)	0.0063	36 (39.1)
No. (%) of EU-TIRADS 5 nodules	7 (11.1)	18 (48.7)	<0.0001	25 (25.0)	8 (7.5)	10 (41.7)	<0.0001	18 (13.7)	6 (13.3)	33 (70.2)	<0.0001	39 (42.4)

**Table 2:** Values of elastographic measures in the examined groups of nodules (AUS-nuclear, AUS-other, and nodules with unequivocal cytology—Bethesda 2 and 6) in the relation to the definite histopathological diagnosis (benign vs. malignant).

SWE Measure	Size of Nodules	AUS-Nuclear			AUS-Other			Unequivocal Cytology		
		Benign	Malignant	*p*	Benign	Malignant	*p*	Benign	Malignant	*p*
Mean Ewhole ± SD [m/s]	>1 cm	2.8 ± 0.6	3.3 ± 1.0	0.0480	2.7 ± 0.6	3.4 ± 1.0	0.0043	3.1 ± 0.8	3.9 ± 1.3	0.0027
	≤1 cm	3.1 ± 0.7	3.1 ± 0.7	0.3738	3.0 ± 0.8	3.2 ± 0.9	0.4636	3.5 ± 0.5	4.6 ± 1.2	0.0257
Mean Estiffest ± SD [m/s]	>1 cm	3.4 ± 0.7	4.3 ± 1.3	0.0276	3.3 ± 0.8	4.0 ± 1.1	0.0262	3.7 ± 0.7	4.7 ± 1.5	0.0023
	≤1 cm	3.4 ± 0.7	3.5 ± 0.8	0.9891	3.3 ± 0.9	3.7 ± 0.9	0.1729	3.7 ± 0.4	5.1 ± 1.2	0.0054

**Table 3:** Diagnostic effectiveness of SWE metrics, EU-TIRADS class, and rFNA in AUS-nuclear, AUS-other, and cytologically unequivocal nodules (note: repeat FNA was not performed in the case of unequivocal diagnosis of malignancy).

Size of Nodule	Feature	AUC [CI95%] *p*	Cut-Off [m/s]	ACC [%]	SEN [%]	SPC [%]	PPV [%]	NPV [%]	Positive Nodules [%]
	**AUS-nuclear**								
>1 cm	Ewhole	0.634 [0.440–0.828] *p* = 0.1758	3.43	75.0	50.0	82.6	46.7	84.4	25.0
	Estiffest	0.696 [0.525–0.867] *p* = 0.0244	4.31	81.7	50.0	91.3	63.6	85.7	18.3
	EU-TIRADS	0.745 [0.568–0.922] *p* = 0.0066	5	86.7	57.1	95.7	80.0	88.0	16.7
	rFNA	0.849 [0.730–0.988] *p* = 0.0000	5	90.0	71.4	95.7	83.3	91.7	20.0
≤1 cm	Ewhole	0.416 [0.224–0.607] *p* = 0.3886	2.61	60.0	87.0	23.5	60.6	57.1	82.5
	Estiffest	0.503 [0.317–0.688] *p* = 0.9785	4.34	52.5	17.4	100.0	100.0	47.2	10.0
	EU-TIRADS	0.710 [0.550–0.870] *p* = 0.0101	5	65.0	43.5	94.1	90.9	55.2	27.5
	rFNA	0.921 [0.831–1.000] *p* = 0.0000	5	87.5	87.0	88.2	90.9	83.3	55.0
	**AUS-other**								
>1 cm	Ewhole	0.752 [0.602–0.902] *p* = 0.0010	3.22	83.8	61.5	88.1	50.0	92.2	20.0
	Estiffest	0.697 [0.538–0.856] *p* = 0.0154	4.09	81.3	46.2	88.1	42.9	89.4	17.5
	EU-TIRADS	0.715 [0.552–0.879] *p* = 0.0098	5	87.5	30.8	98.5	80.0	88.0	6.3
	rFNA	0.763 [0.602–0.941] *p* = 0.0013	5	91.3	46.2	100.0	100.0	90.5	7.5
≤1 cm	Ewhole	0.574 [0.391–0.757] *p* = 0.4296	2.64	47.1	81.8	37.5	26.5	88.2	66.7
	Estiffest	0.636 [0.470–0.803] *p* = 0.1084	2.67	45.1	100.0	30.0	28.2	100.0	76.5
	EU-TIRADS	0.736 [0.577–0.896] *p* = 0.0037	5	76.5	54.5	82.5	46.2	86.8	25.5
	rFNA	0.830 [0.655–1.000] *p* = 0.0002	5	92.2	63.6	100.0	100.0	90.9	13.7
	**unequivocal cytology**								
>1 cm	Ewhole	0.744 [0.582–0.905] *p* = 0.0031	3.40	80.4	75.0	83.3	71.4	85.7	37.5
	Estiffest	0.748 [0.596–0.900] *p* = 0.0014	4.19	82.1	65.0	91.7	81.3	82.5	28.6
	EU-TIRADS	0.725 [0.578–0.872] *p* = 0.0027	5	76.8	50.0	91.7	76.9	76.7	23.2
≤1 cm	Ewhole	0.753 [0.599–0.907] *p* = 0.0013	4.28	72.2	63.0	100.0	100.0	47.4	47.2
	Estiffest	0.805 [0.678–0.951] *p* = 0.0000	4.36	77.8	70.4	100.0	100.0	52.9	52.8
	EU-TIRADS	0.809 [0.603–0.100] *p* = 0.0033	5	80.6	85.2	66.7	88.5	60.0	72.2

**Table 4:** Results of single- and multivariate logistic regression analysis in AUS-nuclear, AUS-other, and cytologically unequivocal nodules.

Size of Nodule	Type of Logistic Analysis	Feature	OR [95%CI] *p*		
			AUS-Nuclear	AUS-Other	Unequivocal Cytology
>1 cm	Univariate analysis	Ewhole	2.2 [1.0–4.8] *p* = 0.0524	3.5 [1.5–7.9] *p* = 0.0031	2.1 [1.1–4.0] *p* = 0.0192
		Estiffest	2.5 [1.2–5.0] *p* = 0.0133	2.5 [1.3–5.1] *p* = 0.0080	2.5 [1.3–4.6] *p* = 0.0049
		EU-TIRADS	5.3 [1.8–15.6] *p* = 0.0027	6.5 [1.7–24.4] *p* = 0.0051	4.8 [1.6–14.1] *p* = 0.0042
		rFNA	6.5 [2.7–15.8] *p* = 0.0000	5.0 [2.0–12.3] *p* = 0.0006	not applicable
	Multivariate analysis	Ewhole	-	-	
		Estiffest	-	-	2.1 [1.1–4.0] *p* = 0.029
		EU-TIRADS	-	5.1 [1.0–25.3] *p* = 0.044	3.4 [1.3–10.4], *p* = 0.029
		rFNA	6.5 [2.7–15.8] *p* < 0.000	5.2 [1.7–15.9] *p* = 0.004	not applicable
≤1 cm	Univariate analysis	Ewhole	NS	NS	3.1 [1.1–8.3] *p* = 0.0272
		Estiffest	NS	NS	4.0 [1.3–12.4] *p* = 0.0153
		EU-TIRADS	5.7 [1.2–11.1] *p* = 0.0216	5.2 [1.4–18.9] *p* = 0.0114	8.2 [2.2–30.0] *p* = 0.0015
		rFNA	5.4 [2.3–13.0] *p* = 0.0001	4.3 [1.8–10.3] *p* = 0.0010	not applicable
	Multivariate analysis	Ewhole	-	-	-
		Estiffest	-	-	-
		EU-TIRADS	-	-	8.2 [2.2–30.0] *p* = 0.001
		rFNA	7.2 [2.5–20.3] *p* < 0.000	4.3 [1.8–10.3] *p* = 0.001	not applicable

**Table 5:** Usefulness of the addition of Estiffest analysis in the AUS-nuclear group and Ewhole in the AUS-other group to the standard EU-TIRADS/repeat FNA evaluation for nodules > 1 cm (all other possible combinations of SWE metrics with EU-TIRADS/repeat FNA evaluation for nodules > 1 cm and smaller nodules are presented in Tables S3 and S4 in Supplementary Materials).

Size of Nodules	Feature	AUC [CI95%] *p*	ACC [%]	SEN [%]	SPC [%]	PPV [%]	NPV [%]	Positive Nodules [%]
>1 cm	**AUS-nuclear**							
	EU-TIRADS or Estiffest	0.792 [0.641–0.927] *p* = 0.0002	83.3	71.4	87.0	62.5	90.9	26.7
	EU-TIRADS or rFNA	0.849 [0.714–0.985] *p* = 0.0000	88.3	78.6	91.3	73.3	93.3	25.0
	EU-TIRADS or rFNA or Estiffest	0.842 [0.717–0.966] *p* = 0.0000	83.3	85.7	82.6	60.0	95.0	33.3
	rFNA or Estiffest	0.828 [0.690–0.966] *p* = 0.0000	85.0	78.6	87.0	64.7	93.0	28.3
	**AUS-other**							
	EU-TIRADS or Ewhole	0.741 [0.574–0.907] *p* = 0.0046	82.5	61.5	86.6	47.1	92.1	21.2
	EU-TIRADS or rFNA	0.762 [0.586–0.938] *p* = 0.0035	91.3	53.8	98.5	87.5	91.7	10.0
	EU-TIRADS or rFNA or Ewhole	0.741 [0.574–0.907] *p* = 0.0046	82.5	61.5	86.6	47.1	92.1	21.2
	rFNA or Ewhole	0.748 [0.582–0.914] *p* = 0.0035	83.8	61.5	88.1	50.0	92.2	20.0

## Data Availability

The original contributions presented in this study are included in the article/Supplementary Material. Further inquiries can be directed to the corresponding author(s).

## Supplementary Materials

The following supporting information can be downloaded at: https://www.mdpi.com/article/10.3390/cancers18172737/s1, Figure S1: Diagram illustrating how the patients were assigned to the study groups; Table S1: Types of cancers in examined groups of nodules; Table S2: Outcomes of repeat FNA in AUS-nuclear and AUS-other groups in relation to the nodule’s size and the definite histopathological diagnosis (benign vs. malignant); Table S3: Diagnostic effectiveness of the combined assessment of SWE measures, EU-TIRADS class, and repeat FNA in AUS-nuclear or AUS-other nodules > 1 cm; Table S4: Diagnostic effectiveness of the combined assessment of SWE measures, EU-TIRADS class, and repeat FNA in AUS-nuclear or AUS-other nodules ≤ 1 cm.

## Abbreviations

The following abbreviations are used in this manuscript:

AUS	atypia of undetermined significance
AUS-nuclear	atypia of undetermined significance with nuclear atypia
AUS-other	atypia of undetermined significance with other patterns of atypia
BSRTC	Bethesda System for Reporting Thyroid Cytopathology
EU-TIRADS	European Thyroid Imaging and Reporting Data System
FN	follicular neoplasm
FNA	fine-needle aspiration biopsy
FTC	follicular thyroid carcinoma
MN	malignant neoplasm
NIFTP	noninvasive follicular thyroid neoplasm with papillary-like nuclear features
NPV	negative predictive value
OR	odds ratio
PPV	positive predictive value
PTC	papillary thyroid carcinoma
SM	suspicious for malignancy

## References

[1] Ali S.Z., Baloch Z.W., Cochand-Priollet B., Schmitt F.C., Vielh P., VanderLaan P.A. (2023). The 2023 Bethesda System for Reporting Thyroid Cytopathology. Thyroid.

[2] Haugen B.R., Alexander E.K., Bible K.C., Doherty G.M., Mandel S.J., Nikiforov Y.E., Pacini F., Randolph G.W., Sawka A.M., Schlumberger M. (2016). 2015 American Thyroid Association Management Guidelines for Adult Patients with Thyroid Nodules and Differentiated Thyroid Cancer: The American Thyroid Association Guidelines Task Force on Thyroid Nodules and Differentiated Thyroid Cancer. Thyroid.

[3] Durante C., Hegedüs L., Czarniecka A., Paschke R., Russ G., Schmitt F., Soares P., Solymosi T., Papini E. (2023). 2023 European Thyroid Association Clinical Practice Guidelines for thyroid nodule management. Eur. Thyroid J..

[4] Vuong H.G., Ngo H.T.T., Bychkov A., Jung C.K., Vu T.H., Lu K.B., Kakudo K., Kondo T. (2020). Differences in surgical resection rate and risk of malignancy in thyroid cytopathology practice between Western and Asian countries: A systematic review and meta-analysis. Cancer Cytopathol..

[5] Ooi L.Y., Nga M.E. (2020). Atypia of undetermined significance/follicular lesion of undetermined significance: Asian vs. non-Asian practice, and the Singapore experience. Gland Surg..

[6] Gan T.R., Nga M.E., Lum J.H., Wong W.M., Tan W.B., Parameswaran R., Ngiam K.Y. (2017). Thyroid cytology-nuclear versus architectural atypia within the “Atypia of undetermined significance/follicular lesion of undetermined significance” Bethesda category have significantly different rates of malignancy. Cancer Cytopathol..

[7] Słowińska-Klencka D., Popowicz B., Duda-Szymańska J., Klencki M. (2025). Thyroid Nodules with Nuclear Atypia of Undetermined Significance (AUS-Nuclear) Hold a Two-Times-Higher Risk of Malignancy than AUS-Other Nodules Regardless of EU-TIRADS Class of the Nodule or Borderline Tumor Interpretation. Cancers.

[8] Swan K.Z., Nielsen V.E., Bonnema S.J. (2021). Evaluation of thyroid nodules by shear wave elastography: A review of current knowledge. J. Endocrinol. Investig..

[9] Bamber J., Cosgrove D., Dietrich C.F., Fromageau J., Bojunga J., Calliada F., Cantisani V., Correas J.M., D’Onofrio M., Drakonaki E.E. (2013). EFSUMB guidelines and recommendations on the clinical use of ultrasound elastography. Part 1: Basic principles and technology. Ultraschall Med..

[10] Shiina T., Nightingale K.R., Palmeri M.L., Hall T.J., Bamber J.C., Barr R.G., Castera L., Choi B.I., Chou Y.H., Cosgrove D. (2015). WFUMB guidelines and recommendations for clinical use of ultrasound elastography: Part 1: Basic principles and terminology. Ultrasound Med. Biol..

[11] Ferraioli G., Barr R.G., Farrokh A., Radzina M., Cui X.W., Dong Y., Rocher L., Cantisani V., Polito E., D’Onofrio M. (2022). How to perform shear wave elastography. Part I. Med. Ultrason..

[12] Săftoiu A., Gilja O.H., Sidhu P.S., Dietrich C.F., Cantisani V., Amy D., Bachmann-Nielsen M., Bob F., Bojunga J., Brock M. (2019). The EFSUMB Guidelines and Recommendations for the Clinical Practice of Elastography in Non-Hepatic Applications: Update 2018. Ultraschall Med..

[13] Cosgrove D., Barr R., Bojunga J., Cantisani V., Chammas M.C., Dighe M., Vinayak S., Xu J.M., Dietrich C.F. (2017). WFUMB Guidelines and Recommendations on the Clinical Use of Ultrasound Elastography: Part 4. Thyroid. Ultrasound Med. Biol..

[14] Szczepanek-Parulska E., Woliński K., Stangierski A., Gurgul E., Biczysko M., Majewski P., Rewaj-Łosyk M., Ruchała M. (2013). Comparison of diagnostic value of conventional ultrasonography and shear wave elastography in the prediction of thyroid lesions malignancy. PLoS ONE.

[15] Swan K.Z., Bonnema S.J., Jespersen M.L., Nielsen V.E. (2019). Reappraisal of shear wave elastography as a diagnostic tool for identifying thyroid carcinoma. Endocr. Connect..

[16] Cantisani V., David E., Grazhdani H., Rubini A., Radzina M., Dietrich C.F., Durante C., Lamartina L., Grani G., Valeria A. (2019). Prospective Evaluation of Semiquantitative Strain Ratio and Quantitative 2D Ultrasound Shear Wave Elastography (SWE) in Association with TIRADS Classification for Thyroid Nodule Characterization. Ultraschall Med..

[17] Yi A.J., Yang W.W., Cui X.W., Dietrich C.F., Wang B. (2024). The value of quantitative and a new qualitative color pattern shear wave elastography for the differentiation of ACR TI-RADS 4 or 5 category thyroid nodules measuring ≤10 mm. Front. Endocrinol..

[18] Filho R.H.C., Pereira F.L., Iared W. (2020). Diagnostic Accuracy Evaluation of Two- Dimensional Shear Wave Elastography in the Differentiation Between Benign and Malignant Thyroid Nodules: Systematic Review and Meta-Analysis. J. Ultrasound Med..

[19] Jarząb B., Dedecjus M., Lewiński A., Adamczewski Z., Bakuła-Zalewska E., Bałdys-Waligórska A., Barczyński M., Biskup-Frużyńska M., Bobek-Billewicz B., Bossowski A. (2022). Diagnosis and treatment of thyroid cancer in adult patients—Recommendations of Polish Scientific Societies and the National Oncological Strategy. Endokrynol. Pol..

[20] Baloch Z.W., Asa S.L., Barletta J.A., Ghossein R.A., Juhlin C.C., Jung C.K., LiVolsi V.A., Papotti M.G., Sobrinho-Simões M., Tallini G. (2022). Overview of the 2022 WHO Classification of Thyroid Neoplasms. Endocr. Pathol..

[21] Chang N., Zhang X., Wan W., Zhang C., Zhang X. (2018). The Preciseness in Diagnosing Thyroid Malignant Nodules Using Shear-Wave Elastography. Med. Sci. Monit..

[22] Bardet S., Ciappuccini R., Pellot-Barakat C., Monpeyssen H., Michels J.J., Tissier F., Blanchard D., Menegaux F., de Raucourt D., Lefort M. (2017). Shear Wave Elastography in Thyroid Nodules with Indeterminate Cytology: Results of a Prospective Bicentric Study. Thyroid.

[23] Liu X., Medici M., Kwong N., Angell T.E., Marqusee E., Kim M.I., Larsen P.R., Cho N.L., Nehs M.A., Ruan D.T. (2016). Bethesda Categorization of Thyroid Nodule Cytology and Prediction of Thyroid Cancer Type and Prognosis. Thyroid.

[24] Sun C.F., Wu D., Sun J.Y., Hou Y.L., Xin J.W., Ru X.C., Zhang Y.F. (2025). Diagnostic performance of ultrasound elastography alone and in combination with conventional ultrasonography for Bethesda III-IV thyroid nodules: A systematic review and meta-analysis. Quant. Imaging Med. Surg..

[25] Samir A.E., Dhyani M., Anvari A., Prescott J., Halpern E.F., Faquin W.C., Stephen A. (2015). Shear-Wave Elastography for the Preoperative Risk Stratification of Follicular-patterned Lesions of the Thyroid: Diagnostic Accuracy and Optimal Measurement Plane. Radiology.

[26] Azizi G., Keller J.M., Mayo M.L., Piper K., Puett D., Earp K.M., Malchoff C.D. (2018). Shear wave elastography and Afirma™ gene expression classifier in thyroid nodules with indeterminate cytology: A comparison study. Endocrine.

[27] Moraes P.H.M., Takahashi M.S., Vanderlei F.A.B., Schelini M.V., Chacon D.A., Tavares M.R., Chammas M.C. (2021). Multiparametric Ultrasound Evaluation of the Thyroid: Elastography as a Key Tool in the Risk Prediction of Undetermined Nodules (Bethesda III and IV)-Histopathological Correlation. Ultrasound Med. Biol..

[28] Chambara N., Lo X., Chow T.C.M., Lai C.M.S., Liu S.Y.W., Ying M. (2022). Combined Shear Wave Elastography and EU TIRADS in Differentiating Malignant and Benign Thyroid Nodules. Cancers.

[29] Appanraj P., Kaur J., George N.A., Chinniah P.K. (2024). Role of Ultrasound Elastography in Evaluating Suspicious Thyroid Nodules. Indian. J. Surg. Oncol..

[30] Celletti I., Fresilli D., De Vito C., Bononi M., Cardaccio S., Cozzolino A., Durante C., Grani G., Grimaldi G., Isidori A.M. (2021). TIRADS, SRE and SWE in indeterminate thyroid nodule characterization: Which has better diagnostic performance?. Radiol. Med..

[31] Qiu Y., Xing Z., Yang Q., Luo Y., Ma B. (2023). Diagnostic performance of shear wave elastography in thyroid nodules with indeterminate cytology: A systematic review and meta-analysis. Heliyon.

[32] Zhang W.B., Li J.J., Chen X.Y., He B.L., Shen R.H., Liu H., Chen J., He X.F. (2020). SWE combined with ACR TI-RADS categories for malignancy risk stratification of thyroid nodules with indeterminate FNA cytology. Clin. Hemorheol. Microcirc..

[33] Cheng K.L., Lai P.H., Su C.L., Baek J.H., Lee H.L. (2023). Impact of Region-of-Interest Size on the Diagnostic Performance of Shear Wave Elastography in Differentiating Thyroid Nodules. Cancers.

[34] Gao X.Q., Ma Y., Peng X.S., Wang L.L., Li H.X., Zheng X.L., Liu Y. (2022). Diagnostic performance of C-TIRADS combined with SWE for the diagnosis of thyroid nodules. Front. Endocrinol..

[35] Dobruch-Sobczak K., Zalewska E.B., Gumińska A., Słapa R.Z., Mlosek K., Wareluk P., Jakubowski W., Dedecjus M. (2016). Diagnostic Performance of Shear Wave Elastography Parameters Alone and in Combination with Conventional B-Mode Ultrasound Parameters for the Characterization of Thyroid Nodules: A Prospective, Dual-Center Study. Ultrasound Med. Biol..

[36] Latia M., Bena A., Moisa-Luca L., Bunceanu Ș., Stoian D. (2025). Shear wave elastography for thyroid nodule evaluation in patients with chronic autoimmune thyroiditis. Endocrine.

[37] Wang D., He Y.P., Zhang Y.F., Liu B.J., Zhao C.K., Fu H.J., Wei Q., Xu H.X. (2017). The diagnostic performance of shear wave speed (SWS) imaging for thyroid nodules with elasticity modulus and SWS measurement. Oncotarget.

[38] Qi W.H., Jin K., Cao L.L., Peng M., He N.A., Zhan X.L., Yang Y., Guo Y.Y., Cui X.W., Jiang F. (2023). Diagnostic performance of a new two-dimensional shear wave elastography expression using siemens ultrasound system combined with ACR TI-RADS for classification of benign and malignant thyroid nodules: A prospective multi-center study. Heliyon.

[39] Liu B., Liang J., Zheng Y., Xie X., Huang G., Zhou L., Wang W., Lu M. (2015). Two-dimensional shear wave elastography as promising diagnostic tool for predicting malignant thyroid nodules: A prospective single-centre experience. Eur. Radiol..

[40] Kim H., Kim J.A., Son E.J., Youk J.H. (2013). Quantitative assessment of shear-wave ultrasound elastography in thyroid nodules: Diagnostic performance for predicting malignancy. Eur. Radiol..

[41] Zhou Y., Li R., Zheng W., Liu Z., Yang J., Wang L. (2026). SWE, C-TIRADS and AI-assisted diagnostic systems in distinguishing thyroid nodules of different lesion size: Which has better diagnostic performance?. Front. Endocrinol..

[42] Xue J.P., Kang X.Y., Miao J.W., Zhang Y.X., Li H.Z., Yao F.C., Kang C.S. (2022). Analysis of the Influence of Thyroid Nodule Characteristics on the Results of Shear Wave Elastography. Front. Endocrinol..

[43] Wang F., Chang C., Chen M., Gao Y., Chen Y.L., Zhou S.C., Li J.W., Zhi W.X. (2018). Does Lesion Size Affect the Value of Shear Wave Elastography for Differentiating Between Benign and Malignant Thyroid Nodules?. J. Ultrasound Med..

[44] Duan S.B., Yu J., Li X., Han Z.Y., Zhai H.Y., Liang P. (2016). Diagnostic value of two-dimensional shear wave elastography in papillary thyroid microcarcinoma. Onco Targets Ther..

[45] Bora Makal G., Aslan A. (2021). The Diagnostic Value of the American College of Radiology Thyroid Imaging Reporting and Data System Classification and Shear-Wave Elastography for the Differentiation of Thyroid Nodules. Ultrasound Med. Biol..

[46] Kara T., Ateş F., Durmaz M.S., Akyürek N., Durmaz F.G., Özbakır B., Öztürk M. (2020). Assessment of thyroid gland elasticity with shear-wave elastography in Hashimoto’s thyroiditis patients. J. Ultrasound.

