# Supplementary material for: Shear Wave Elastography Adds to the Sensitivity of Cancer Diagnosis in AUS-Nuclear and AUS-Other Nodules > 1 cm, but It Cannot Replace TIRADS Assessment and Repeat FNA
Source: Cancers (Basel). 2026 Aug 23;18(17):2737. doi: 10.3390/cancers18172737 (PMC13565322; doi:10.3390/cancers18172737)

Table S1. Types of cancers in examined groups of nodules.

Type of cancer	AUS-nuclear No. (%)	AUS-other No. (%)	unequivocal cytology No. (%)
PTC	27(73.0)	15 (62.5)	45 (95.7)
FTC	3 (8.1)	3 (12.5)	-
MTC	1 (2.7)	2	-
PDTC	-	-	1 (2.1)
NIFTP	5 (13.5)	1	-
TT-	1 (2.7)	2	-
ST	-	1 (4.2) a	1 (2.1) b
All	37	24	47

a - clear cell renal cell carcinoma; b - malignant melanoma

PTC—papillary thyroid carcinoma, FTC follicular thyroid carcinoma, MTC—medullary thyroid carcinoma, PDTC—poorly differentiated thyroid carcinoma, NIFTP - non-invasive follicular thyroid neoplasm with papillary like nuclear features, TT-UMP—thyroid tumor of uncertain malignant potential, ST—secondary tumor

Table S2. Outcomes of repeat FNA in AUS-nuclear and AUS-other groups in relation to the nodule's size and the definite histopathological diagnosis (benign vs. malignant).

rFNA category	AUS-nuclear number (%)				AUS-other number (%)			
	> 1 cm		≤ 1 cm		> 1 cm		≤ 1 cm	
	**benign**	**malignant**	**benign**	**malignant**	**benign**	**malignant**	**benign**	**malignant**
I	0	0	1 (5.9)	0	6 (9.0)	0	5 (12.5)	1 (9.1)
II	3 (6.5)	0	8 (47.0)	0	21 (31.3)	2 (15.4)	13 (32.5)	0
III	41 (89.1)	4 (28.6)	6 (35.3)	3 (13.0)	38 (56.7)	5 (38.5)	19 (47.5)	3 (27.3)
IV	0	0	0	0	2 (3.0)	0	3 (7.5)	0
V	2 (4.3)	7 (50.0)	2 (11.8)	16 (69.6)	0	5 (38.5)	0	5 (45.5)
VI	0	3 (21.4)	0	4 (17.4)	0	1 (7.7)	0	2 (18.2)
All	46 (100)	14 (100)	17 (100)	23 (100)	67 (100)	13 (100)	40 (100)	11 (100)

Table S3. Diagnostic effectiveness of the combined assessment of SWE measures, EU-TIRADS class and repeat FNA in AUS-nuclear or AUS-other nodules > 1 cm

Size of nodule	parameter	Ewhole or Estiffest	EU-TIRADS or Ewhole	EU-TIRADS or Estiffest	EU-TIRADS or Ewhole or Estiffest	rFNA or EU-TIRADS	rFNA or Ewhole	rFNA or Estiffest	rFNA or Ewhole or Estiffest	rFNA or EU-TIRADS or Ewhole	rFNA or EU-TIRADS or Estiffest	rFNA or EU-TIRADS or Ewhole or Estiffest
> 1 cm	**AUS nuclear**											
	AUC	0.699	0.713	0.792	0.748	0.849	0.795	0.828	0.784	0.798	0.842	0.798
	ACC %	76.7	75.0	83.3	76.7	88.3	80.0	85.0	78.3	76.7	83.3	76.7
	SEN %	57.1	64.3	71.4	71.4	78.6	78.6	78.6	78.6	85.7	85.7	85.7
	SPC %	82.6	78.3	87.0	78.3	91.3	80.4	87.0	78.3	73.9	82.6	73.9
	PPV %	50.0	47.4	62.5	50.0	73.3	55.0	64.7	52.4	50.0	60.0	50.0
	NPV %	86.4	87.8	90.9	90.0	93.3	92.5	93.0	92.3	94.4	95.0	94.4
	positive nodules	26.7	31.7	26.7	33.3	25.0	33.3	28.3	35.0	40.0	33.3	40.0
	**AUS-other**											
	AUC	0.748	0.741	0.664	0.733	0.762	0.748	0.748	0.748	0.741	0.741	0.733
	ACC %	83.8	82.5	80.0	81.3	91.3	83.8	83.8	83.8	82.5	82.5	81.3
	SEN %	61.5	61.5	46.2	61.5	53.8	61.5	61.5	61.5	61.5	61.5	61.5
	SPC %	88.1	86.6	86.6	85.1	98.5	88.1	88.1	88.1	86.6	86.6	85.1
	PPV %	50.0	47.1	40.0	44.4	87.5	50.0	50.0	50.0	47.1	47.1	44.4
	NPV %	92.2	92.1	89.2	91.9	91.7	92.2	92.2	92.2	92.1	92.1	91.9
	positive nodules	20.0	21.2	18.7	22.5	10.0	20.0	20.0	20.0	21.2	21.2	22.5

Table S4. Diagnostic effectiveness of the combined assessment of SWE measures, EU-TIRADS class and repeat FNA in AUS-nuclear or AUS-other nodules ≤ 1 cm.

Size of nodule	parameter	Ewhole or Estiffest	EU-TIRADS or Ewhole	EU-TIRADS or Estiffest	EU-TIRADS or Ewhole or Estiffest	rFNA or EU-TIRADS	rFNA or Ewhole	rFNA or Estiffest	rFNA or Ewhole or Estiffest	rFNA or EU-TIRADS or Ewhole	rFNA or EU-TIRADS or Estiffest	rFNA or EU-TIRADS or Ewhole or Estiffest
≤ 1 cm	**AUS nuclear**											
	AUC	0.552	0.574	0.753	0.574	0.847	0.588	0.898	0.588	0.588	0.751	0.588
	ACC %	60.0	62.5	72.5	62.5	85.0	65.0	90.0	65.0	65.0	77.5	65.0
	SEN %	87.0	91.3	56.5	91.3	87.0	100.0	91.3	100.0	100.0	91.3	100.0
	SPC %	23.5	23.5	94.1	23.5	82.4	17.6	88.2	17.6	17.6	58.8	17.6
	PPV %	60.6	61.8	92.9	61.8	87.0	62.2	91.3	62.2	62.2	75.0	62.2
	NPV %	57.1	66.7	61.5	66.7	82.4	100.0	88.2	100.0	100.0	83.3	100.0
	positive nodules	82.5	85.0	35.0	85.0	57.5	92.5	57.5	92.5	92.5	70.0	92.5
	**AUS-other**											
	AUC	0.650	0.605	0.625	0.625	0.776	0.642	0.650	0.650	0.650	0.625	0.625
	ACC %	45.1	43.1	41.2	41.2	80.4	49.0	45.1	45.1	45.1	41.2	41.2
	SEN %	100.0	90.9	100.0	100.0	72.7	90.9	100.0	100.0	100.0	100.0	100.0
	SPC %	30.0	30.0	25.0	25.0	82.5	37.5	30.0	30.0	30.0	25.0	25.0
	PPV %	28.2	26.3	26.8	26.8	53.3	28.6	28.2	28.2	28.2	26.8	26.8
	NPV %	100.0	92.3	100.0	100.0	91.7	93.8	100.0	100.0	100.0	100.0	100.0
	positive nodules	76.5	74.5	80.4	80.4	29.4	68.6	76.5	76.5	76.5	80.4	80.4

Figure S1. Diagram illustrating how the patients were assigned to the study groups.

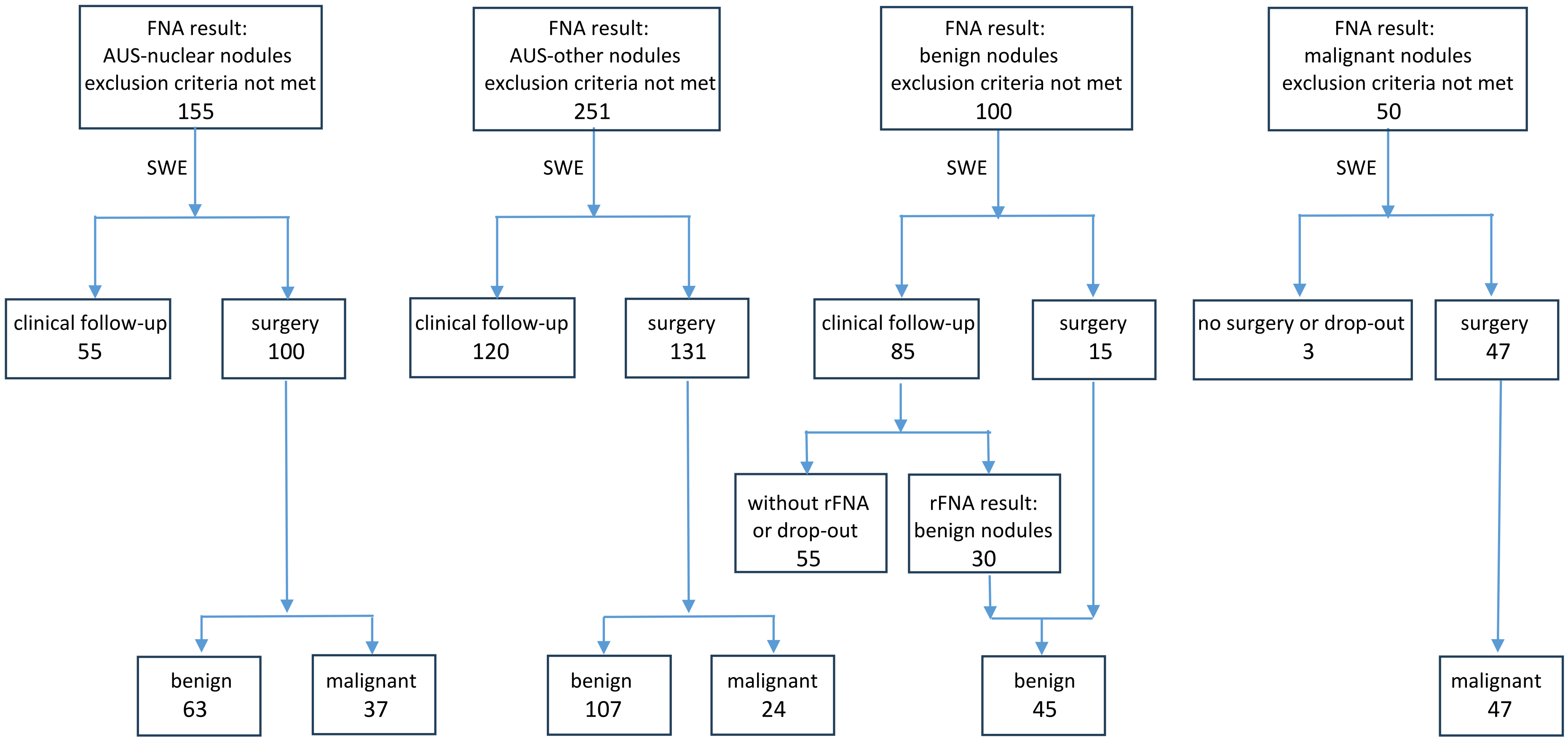

Supplement: Supplementary file 1 [file cancers-18-02737-s001.zip › cancers-4462918-supplementary.pdf]
